# The Future of the Public Health Service

**Published:** 1909-09-04

**Authors:** 


### THE FUTURE OF THE PUBLIC HEALTH SERVICE.

Much has been written already concerning the rapid progress and the great developments which have been made in preventive medicine during the last decade. Such retrospection and introspection have been of value in showing the present position of this important service. During the past twelve months several questions of the highest importance have come prominently before the medical profession—and, indeed, before the general public—which render it advisable and interesting to forecast the future of preventive medicine. Some of these most important questions are the majority and the minority reports of the Royal Commission on the Poor Laws, and the defects which have been brought to light as a result of the medical inspection of school children.

Important side-issues have arisen in connection with these questions which require the most earnest consideration and foreshadow great future progress in the public health service. Until recently many of these questions were not considered to be within the province of public health, but there is evident a change of opinion as to the future scope of this branch of medicine, and the old ideas are undergoing considerable modification. For instance, the opinion has been expressed frequently that the public health branch of medicine is concerned with groups of people rather than with individuals. It is probable that the range of public health in the future will necessitate more prominent attention being given to the individual in health or disease than has been given in the past. This modified view has received a great impetus by the reports of the Royal Commission on the Poor Laws issued during February 1909, which form one of the most important Blue Books of modern times. Although there are two different sets of opinions, as shown by the majority and minority reports, both sides have many points in common. Both agree to abolish the direct election of the guardians, and with it the guardians themselves; to abolish the Union area and the general workhouse, substituting for the one a much larger area, and for the other a system of classified institutions; to substitute the name "Public Assistance" for Poor Law; to make the new area the county or the county borough; and to make the Council either itself the authority or directly responsible for appointing the authority.

The majority recommend that the new public assistance authority should be a statutory committee to be appointed by the Council, and that of this one-half may be members of the Council, and the other half are to be outside the Council and to consist of persons experienced in the local administration of public assistance or other cognate work. Thus the presence and help of certain ex-guardians and charity organisation officials would be available.

On the other hand, the minority report recommends that all the work of the existing guardians should be handed over bodily to the Council, and that the Poor Law should be broken up by dividing it amongst the different existing committees for health, education, pensions, etc. For example, they recommend that the provision for (*a*) children of school age and (*b*) the sick and the permanently incapacitated, the infants under school age, and the aged needing institutional care, should be assumed under the direction of the county and county borough councils by (*a*) the education committee, and (*b*) the health committee respectively.

Again, it will be remembered that during the year 1907 the Board of Education approved of the principle of the compulsory medical examination of children in public elementary schools, and that it initiated a medical department under its own auspices for the administration thereof. As a result, a very large number of school medical officers have been appointed throughout the country to carry out the required work. In many localities, in accordance with the recommendation of the Board of Education, the work of medical inspection is being carried out in intimate conjunction with the public health authority, and under the supervision of the medical officer of health. The advantages of this arrangement are very great. Consequently, an immense number of school children have been examined during the year 1908 in various parts of England. This has resulted in the discovery of a considerable number of children suffering from defects which should be remedied, so that they may be enabled to continue in attendance at school without injury to themselves, and with the fullest educational advantages. It will be admitted generally that the accumulation of large volumes of statistics without further steps will not benefit the children. Experience shows that a large number of children who have remediable defects have not received suitable treatment. In some cases apathy, and in other cases poverty of the parents, is the cause.

It is probable that the treatment of many of these children will continue to be ignored unless the cases can be followed up persistently by home visiting, and unless other facilities for treatment can be provided. Therefore each locality will be obliged to consider whether the present available institutions are able to deal with the cases, or whether new organisations should be commenced. Thus it is clear that the work of various health departments in this country may be considerably modified and extended in the near future.

In recent years the work of the public health service has extended from the steps taken to prevent infection, recurrence of nuisances, etc., to a wider sphere, having for its object the prolongation and saving of life. As instances of such extended measures may be mentioned industrial legislation in the prevention of lead poisoning, and in improved conditions of the cotton industry, the administration of the Midwives Act, the Notification of Births Act, the appointment of health visitors, school medical inspection, and the appointment of school nurses.

There is already a close connection between the work of health, education, and Poor Law authorities. But at present there is a considerable amount of overlapping in the work of these three organisations. In future such connection will become much closer, with a diminution in the overlapping. Only by such co-ordination can social efficiency be secured.

To a certain extent the principle of amelioration has been recognised already in dealing with infectious diseases, in provision for blind, deaf and dumb, epileptic, mentally and physically defective children, and also by the administration in many districts of the Education (Provision of Meals) Act. In a few places the treatment of dirty heads, ringworm, and the provision of spectacles have been inaugurated. It is very probable that the above ameliorative measures will in the future become more general, and also that similar measures will extend to other diseases and conditions.

In a circular (No. 576) issued by the Board of Education in November 1907, under the powers of the Education (Administrative Provisions) Act, local education authorities are advised, with no uncertain voice, to formulate schemes of amelioration so that medical inspection, with its records of defects, may be followed by the treatment of such defects. The defects which have been discovered by systematic medical inspection of school children, many of which are remediable, are mainly defects of vision and affections of the eye, ear, teeth, nose, and throat. Since the eye and the ear are the most important avenues of learning, it is necessary that defects of these organs should receive attention with the least possible delay. As to the best method of ensuring this, considerable thought and care must be exercised. Certain lines of action, however, have been indicated already.

In August 1908, the Board of Education issued another circular (596) in which, amongst other things, the arrangements for attending to the health and physical condition of school children are discussed. Included in these arrangements are school clinics, which may serve two purposes, namely, (*a*) for further and more scientific examination of cases in which medical inspection has indicated the existence of defects in a child which cannot conveniently be investigated on the premises of an ordinary public elementary school, (*b*) for the purposes of treatment of defects revealed by inspection. It should be mentioned, however, that before sanctioning the establishment of a school clinic as an "arrangement" under section 13 (1) (b) of the Act, the Board will require detailed information as to the conditions and scope of such a school clinic. For example, it would be necessary to take such precautions that only those children shall be treated in a school clinic for whose treatment adequate provision cannot otherwise be made, whether by the parents or by voluntary associations and institutions, such as hospitals, or through the agency of the Poor Law. Also the Board would require detailed information concerning the precise diseases and defects to be treated, by whom and on what terms and conditions the treatment will be carried out and what will be its extent, and what is the estimated cost of the clinic in respect to buildings and equipment, maintenance, and administration, and treatment, and how it is proposed to meet this cost—out of the rates or otherwise.

Again, it is probable and highly desirable that the public health service of the future should be more closely associated than it has been in the past with the different voluntary organisations which are striving in any town or district to improve the social conditions of its citizens. Even now in certain districts a beginning towards this end has been made. For example, co-operation between a sanitary authority and local Charity Organisation Societies in forming After-care Committees, to obtain work for, and exercise a kindly supervision over, patients who have been discharged from consumption sanatoria with the disease arrested, is already showing beneficial results. Again, After-care Committees containing voluntary workers are being formed with the object of exercising a wise supervision, in co-operation with local education authorities, over children who have left schools established for mentally deficient children. Many other instances could be mentioned to show that in the future the work of the public health official and the voluntary helper will be more closely associated than it has been hitherto; and it is probable that in no branch of medical practice will expansion in the near future be so rapid as in the public health service.

